# Increased expression of Th17 cytokines and interleukin-22 correlates with disease activity in pristane-induced arthritis in rats

**DOI:** 10.1371/journal.pone.0188199

**Published:** 2017-11-28

**Authors:** Bo Wang, Panpan Zhao, Yan Zhou, Liesu Meng, Wenhua Zhu, Congshan Jiang, Linyu Wang, Yongsong Cai, Shemin Lu, Weikun Hou

**Affiliations:** 1 Center for Translational Medicine, the First Affiliated Hospital of Xi’an Jiaotong University, Xi’an, Shaanxi, China; 2 Department of Biochemistry and Molecular Biology, School of Basic Medical Sciences, Xi’an Jiaotong University Health Science Center, Xi’an, Shaanxi, China; 3 Key Laboratory for Tumor Precision Medicine of Shaanxi Province, Xi’an, Shaanxi, China; 4 Department of Dermatology, the First Affiliated Hospital of Xi’an Jiaotong University, Xi’an, Shaanxi, China; 5 Osteonecrosis and Joint Reconstruction Ward, Joint Surgery, Xi’an Honghui Hospital, Xi’an Jiaotong University Health Science Center, Xi’an, Shaanxi, China; University of South Florida St Petersburg, UNITED STATES

## Abstract

The objective of this study was to identify the key changed subtype of T helper cells (Th cells) and their cytokines in pristane-induced arthritis (PIA) in rats. The severity of arthritis was evaluated by body weight, clinical score, the perimeter of ankle and mid-paw and histological assessment of ankle joints. Cytokines of Th1, Th2 and Th17 were determined in the spleen and inguinal lymph nodes at 28 days after pristane injection by real-time qPCR. The mRNA levels of IL-22 receptors, IL-22R1 and IL-22BP, in the spleen were quantified by real-time qPCR. Additionally, IL-22 expression in synovial membrane was detected by Western blotting, and serum IL-22 concentration was determined by ELISA. Correlation between IL-22 concentration and clinical score was analyzed. By screening the cytokines of Th1, Th2 and Th17 expression profile, we found that the mRNA levels of Th17 cytokines were significantly increased in PIA rats. Particularly, a significant increase in the protein expression of IL-22 was determined in synovial membrane and serum from PIA rats, and correlated with clinical score. We conclude that IL-22 expression level was increased and correlated with disease severity, which indicated that IL-22 may play an important role in development of PIA, and was helpful to explorer the pathogenesis of rheumatoid arthritis.

## Introduction

Rheumatoid arthritis (RA) is a common chronic autoimmune inflammatory disorder, which is associated with progressive disability, systemic complications, early death, and socioeconomic costs [1]. RA can cause cartilage and bone damage as well as joint functional disability. The cause of RA is not fully known, however both adaptive and innate autoimmune processes play important roles in the disease pathogenesis [2, 3]. Through the polarization of naive T cells and induction of autoreactive B cells, the innate immune response could further enhance the adaptive immunity [4, 5]. The T helper cells (Th cells), a type of T cell, play an important role by helping to suppress or regulate immune responses in the immune system, particularly in the adaptive immunity. Th cells activate other immune cells by releasing the relational cytokines, and have been confirmed as an important partner in the development of RA and experimental arthritis [6].

Pristane-induced arthritis (PIA) in rats, one of arthritis animal models, is widely used to research the disease pathogenesis because of its high similarity to clinical features of RA, such as having clear acute phase and chronic phase turnover during disease pathogenesis process. It is characterized by joint inflammation, cellular infiltration and destruction of articular cartilage pathologically[7]. Pristane is a natural saturated terpenoid alkane, and known to induce autoimmune diseases in rodents for researching the pathogenesis of rheumatoid arthritis and lupus [7, 8]. PIA is a T-cell-driven arthritis model, which is not dependent on the administration of exogenous antigens [9, 10].Thus, PIA is very suitable for studying the role of cytokines in the pathogenesis of RA.

Th cells, also called auto-reactive effector CD4^+^ T cells, were initially subdivided into two subtypes, Th1 and Th2 cells. They were the host immunity effectors, and their produced cytokines were known as Th1-type cytokines and Th2-type cytokines, respectively. IFN-γ, IL-2 and TNF-α were secreted by Th1 cells, which were critical for the eradication of intracellular pathogens. IL-4, IL-5 and IL-10 were produced by Th2 cells, which were essential for the elimination of extracellular organisms [11]. Th1 were previously considered to be an important drive of autoimmune disease, however this conclusion faces some challenges with inconsistencies [12–14]. The discovery of Th17, a third subtype of Th cells, could bring a new perspective to these challenges.

Th17 cells could produce IL-17 (also known as IL-17A), IL-17F, IL-21, IL-22 and exhibit effector functions distinct from Th1 and Th2 cells [15]. They play an important role in clearance of pathogens, which also induce tissue inflammation and have been associated with the pathogenesis of many experimental autoimmune diseases [16–18]. IL-22 belongs to be a member of IL-10 cytokine family. It consists of 179 amino acid residues, showing 25% sequence identity in human and 22% in mouse with IL-10, an anti-inflammatory and immunosuppressive cytokine [19]. IL-22 primarily targets nonhematopoietic epithelial and stromal cells, where it can promote proliferation and play a role in tissue regeneration [20]. However, it was associated with several conditions involving inflammatory tissue pathology. Recent researches suggested that the expression of IL-22 is abnormal in RA sera and type II collagen-induced arthritis (CIA) mice [21, 22]. Therefore, the strong association of aberrant IL-22 in RA suggests that IL-22 might play a critical role in RA, so it is urgent to develop PIA in rats to a better understanding the regulatory effects of IL-22 in RA pathogenesis.

In the present study, we induced PIA in DA rats to identify differentially expressed cytokines of Th cells, and focused on the significantly changed cytokine in the development process of arthritis. We found that the cytokines of Th17 are enhanced in the spleen and inguinal lymph nodes of PIA rats. Specifically, IL-22 was significantly increased in synovial membrane and aggravated the severity of PIA rats. This research revealed that the signaling pathway of IL-22 may be activated in arthritis pathogenesis. These data would better provide the foundation for RA researching in the future.

## Materials and methods

### Animals

DA rats (originating from Zentralinstitut Fur Versuchstierzucht, Hannover, Germany) were bred in a specific pathogen-free (SPF) animal house of Department of Biochemistry and Molecular Biology, School of Basic Medical Science, Xi’an Jiaotong University Health Science Center. All rats were housed four per polystyrene cage with standard rodent chow and water *ad libitum* under controlled conditions (room temperature 23°C±2°C, room humidity 45% ± 5%, 12h light/dark cycles). The 32 rats at the age of 8 to 12 weeks with a mean weight of 180-200g were randomly divided into the control group and the PIA group matched with sex and age. Rats were euthanized by decapitation following anesthesia with 7% chloral hydrate (0.2ml/100g), and all efforts were made to minimize suffering. All animal experiments were verified and approved by the Institutional Animal Ethics Committee of Xi’an Jiaotong University (permission No. 2013–013).

### Induction and evaluation of arthritis

In PIA group, the arthritis was induced by a single intradermal injection with 300μl of pristane (ACROS Organics, Morris Plains, NJ, USA) at the base of the rat’s tail as described in previous research [8]. The control group was intradermally injected with 300μl phosphate buffered saline. Rats were sacrificed at 28 days after pristane injection. Arthritis development and severity were monitored every two to four days by the body weight, the perimeters of ankle and mid-paw, and a macroscopic scoring system as described previously [7].

Spleens and inguinal lymph nodes of rats were harvested immediately after euthanization, rapidly divided into two parts, and stored at -80°C. Synovial membranes were collected together in each group, and immediately stored at -80°C. The right hind paws were removed and fixed in 4% paraformaldehyde (PFA) solution for one week, then decalcified in 12.5% ethylenediamine tetraacetic acid (EDTA) solution for four weeks, during which the solution was changed every four days. The decalcified samples were embedded in dehydrate paraffin and cut into 5 μm longitudinal sections from the ankle joint center. The sections were stained with hematoxylin and eosin (HE), and observed for pathological changes.

For detection of interested cytokines, blood was collected at 28 days post-immunization, stored at room temperature for 30 min, and centrifuged at 1500×g for 10 min. The sera were equally divided into three samples, and stored at -80°C.

### RNA quantitation

Total RNA was isolated from spleen and inguinal lymph nodes using TRIzol^®^ Reagent (Invitrogen, Carlsbad, CA, USA), and RNA quantitation of samples was measured by using NanoDrop2000 (Thermo, USA). The samples with OD_260/280_ ratios in 1.8–2.0 were reverse-transcribed into cDNA with the First Strand cDNA Synthesis Kit (Fermentas, Burlington, ON, Canada). The cDNA was synthesized from 5μg RNA, and stored at -20°C.

### mRNA expression analysis

Real-time quantitative PCR (RT-qPCR) was performed by CFX96 (Bio-Rad, USA) with FastStart Universal SYBR Geen Master (Roche, USA) for mRNA quantitation of cytokines and their receptors. The reaction conditions were as follows, 95°C 10 min, 95°C 5 s, annealing temperature 30 s, 72°C 30 s, 40 cycles. And the relative gene expression was normalized by β-actin, and calculated by 2^-ΔΔCt^ method. The information on primers, products and annealing temperatures were depicted in Table 1.

**Table 1:** Information of primers for Real-time qPCR.

Gene name	NCBI Accession No.		Sequence (5’-3’)	Size (bp)	Annealing temperature (°C)
*Ifn-γ*	NM_138880.2	Forward Reverse	CCCTCTCTGGCTGTTACTGC TTTCGTGTTACCGTCCTTTTG	149	60
*Tnf-α*	NM_012675.3	Forward Reverse	TCAGCCTCTTCTCATTCCTGC TTGGTGGTTTGCTACGACGTG	203	60
*Tgf-β*	NM_021578.2	Forward Reverse	CTGCTGACCCCCACTGATAC ACGTTTGGGACTGATCCCATT	174	58
*Il-4*	NM_201270.1	Forward Reverse	GTACCGGGAACGGTATCCAC TGGTGTTCCTTGTTGCCGTA	138	58
*Il-10*	NM_012854.2	Forward Reverse	TGCGACGCTGTCATCGATTT GTAGATGCCGGGTGGTTCAA	186	58
*Il-17a*	NM_001106897.1	Forward Reverse	TGGACTCTGAGCCGCAATGA CTCCACCCGGAAAGTGAAGG	189	58
*Il-21*	NM_001108943.2	Forward Reverse	GCACGAAGCTTTTGCCTGTT CACAGGAAGGGCATTTAGCC	161	58
*Il-22*	NM_001191988.1	Forward Reverse	CAACCGCACCTTTATGCTGG ATCCTTGGCTTTGACTCCTCG	103	58
*Il-22ra2*	NM_001003404.1	Forward Reverse	CACTCCATGGTGGGAAACAAAA TCGAACATGGGCTGGTACAT	291	58
*β-actin*	NM_031144.3	Forward Reverse	GAGGGAAATCGTGCGTGAC GCATCGGAACCGCTCATT	157	60

#### Measurement of serum cytokine concentration

Rat sera were collected, and IL-22 was determined using the ELISA development kit (Cusabio, Wuhan, China). Briefly, 100 μl serum or standard were added into the IL-22 antibody-coated plate and incubated for 1 h at 37°C. After adding the biotin-conjugated detecting IL-22 antibody and incubating for 1 h at 37°C, HRP-streptavidin was added and 3,3’-5,5’ tetramethylbenzidin (TMB) was used for development, then stop solution was added to stop reaction. The optical density (OD) value was obtained at the wave of 450 nm using multiskan spectrum (Thermo, USA). The concentration of samples was calculated from standard curve, which was created by the data from the series dilution of recombinant rat IL-22 from zero to 1000pg/ml.

### Western blotting

Total synovial membrane tissue lysates were extracted by using RIPA solution (Beyotime, Suzhou, China) with phenylmethanesulfonyl fluoride (PMSF, Sigma, Germany). The protein concentration for each sample was determined by Pierce^®^ BCA protein assay kit (Thermo, USA).

The lysates protein (40 μg total protein) was prepared and subjected to SDS-PAGE according to standard procedures in the Bio-Rad system. The primary antibody including goat anti-IL22 polyclonal antibody (1:1000 dilution, Abcam, UK) and rabbit anti-β-actin polyclonal antibody (1:1000 dilution, CST, USA) were incubated overnight at 4°C, and the signal was further detected using the secondary antibody of goat anti-rabbit Ig G or mouse anti-goat Ig G conjugated with horseradish peroxidase (HRP) (1:5000, CST, USA) for 2 h at room temperature. Signal intensity was determined by Immobilon^TM^ Western Chemiluminescent HRP Substrate (Millipore, USA). β-actin on the same membrane was used as a loading control. All western blotting analysis were performed in triplicate.

### Statistical analysis

SPSS software (v.19, IBM) was used for statistical analysis. Quantitative data were expressed as means ± SEM. The statistical analysis of differences between experimental groups was performed by using One-way ANOVA or Student’s *t*-test. *P* value < 0.05 was considered statistically significant. Correlation was measured by Spearman’s σ analysis.

## Results

### Establishment of PIA rat model

**Fig 1:**
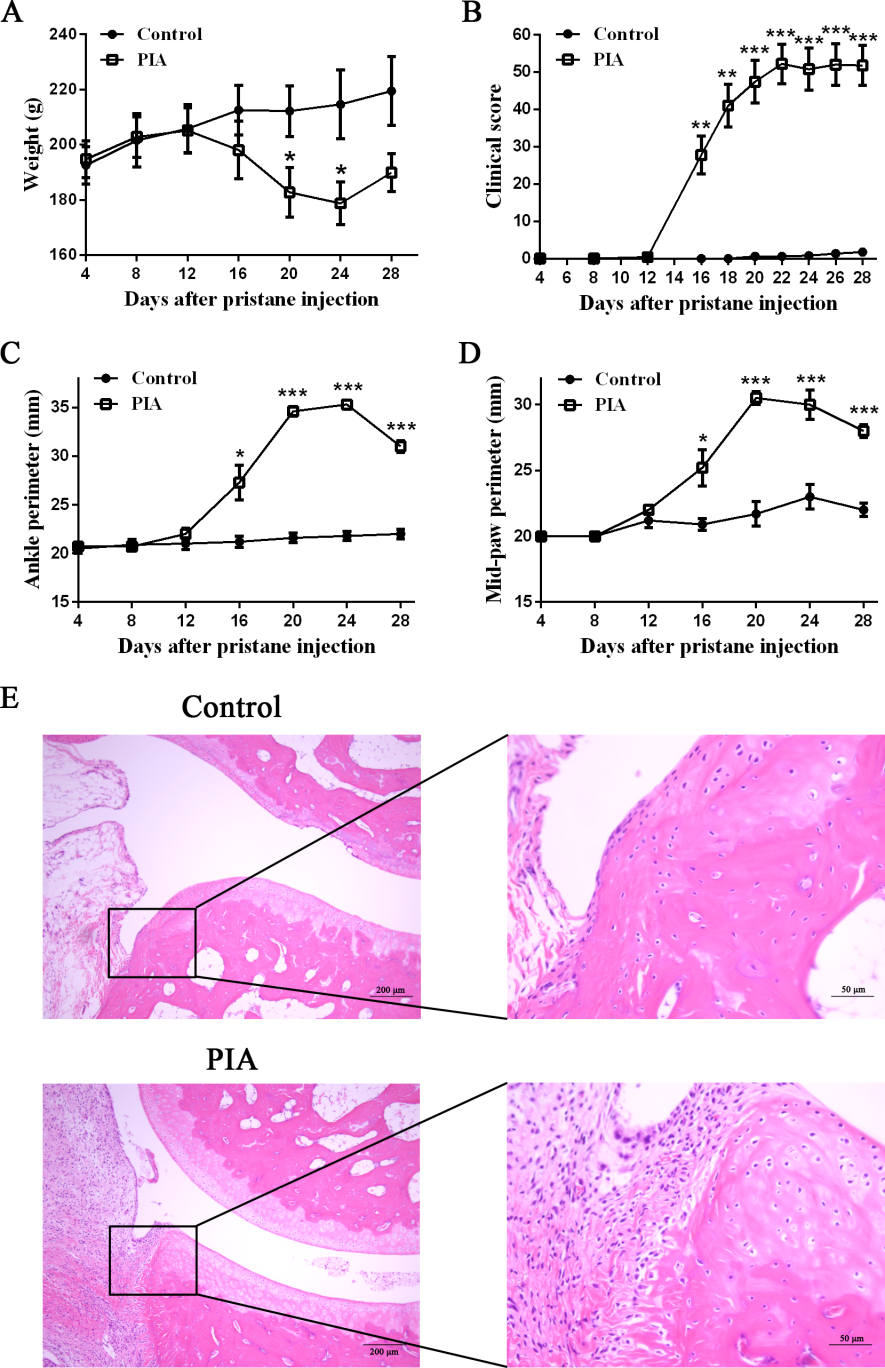
Pristane-induced arthritis induction and evaluation. **(A)** Body weight, **(B)** clinical score, **(C)** ankle perimeter and **(D)** mid-paw perimeter were compared between control and PIA rats (n = 16 per group) after pristane injection. Body weight, ankle and mid-paw perimeters were measured at 4, 8, 12, 16, 20, 24 and 28 days, and clinical score was measured at 4, 8, 12, 16, 18, 20, 22, 24, 26, 28 days after pristane injection. Values are shown as means ± SEM. Levels of significance between the PIA group and control group were calculated by using Mann-Whitney U test (* *P*< 0.05, ** *P*< 0.01 and *** *P*< 0.001). **(E)** Hind ankle joints of control and PIA rats at 28 days were evaluated by H&E staining. The control showed no inflammation and smooth articular surface. PIA rats had infiltration of inflammatory cells, synovial thickening and erosive destruction. Scale bar = 200 μm (left) and 50 μm (right).

As previously reported, arthritis-susceptible DA rats were given a single intradermal injection of pristane known to establish PIA rat model within two weeks [7]. As expected, the PIA group developed acute arthritis within one month. The signs of PIA rat model were evaluated in various aspects, including body weight, clinical score, perimeters of ankle and mid-paw, and pathological changes. The features were observed from 4 days to 28 days after pristane injection. Macroscopic appearance of arthritis could be observed in PIA group at 14 days post-immunization (Fig 1A–1D). In the PIA group, the body weight of rats was reduced at 16 days, and a significant reduction was observed after 20 days (Fig 1A). Consistent with body weight, the clinical score was significantly changed at 16 days, and increased continuously until rats were sacrificed at 28 days (Fig 1B). There were significant differences in the perimeters of ankle and mid-paw between control and PIA group at 16 days, and ankle and mid-paw were exacerbated throughout the course of disease in the rat (Fig 1C and 1D). HE staining sections showed that rat ankle in PIA group revealed infiltration of inflammatory cells, synovial thickening and erosive destruction (Fig 1E). These indicated that PIA rat model was successfully established.

### The expression of Th17 cytokines in the spleen was increased in PIA rat models

**Fig 2:**
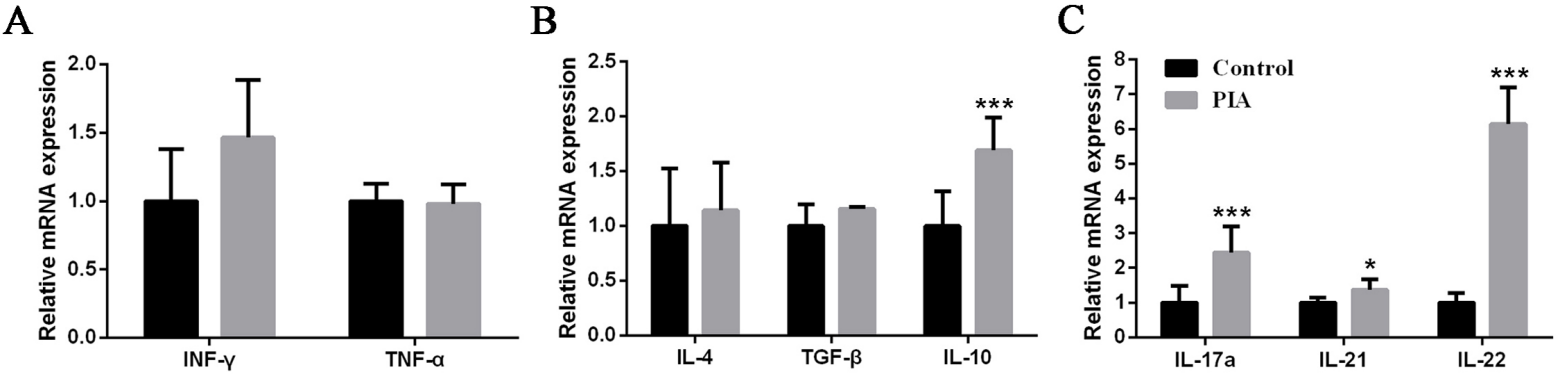
Expression of cytokines in the spleen of PIA rats. **(A)** Expression of IFN-γ and TNF-α, Th1 cytokines, **(B)** expression of Th2 cytokines, IL-4, TGF-β and IL-10, and **(C)** expression of Th17 cytokines, IL-17A, IL-21 and IL-22, at 28 days in rat spleen (n = 10 to 13 for each group) were measured by real-time qPCR. Relative mRNA expression was normalized by β-actin. Values are shown as means ± SEM. Levels of significance between the PIA group and control group were calculated by using Mann-Whitney U test (* *P*< 0.05 and *** *P*< 0.001).

Based on the previous studies, we know that RA is driven by activated Th1 effectors without sufficient Th2 generation to downregulate inflammation, and Th1/Th2 balance is in state of imbalance in animal models [23, 24]. To investigate which cytokine could be involved and significantly changed, the cytokines of Th1, Th2 and Th17 were determined in the spleen between the control and PIA rat model. The spleens were isolated and analyzed for mRNA expression of Th1, Th2 and Th17 cytokines at 28 days. The results showed that IFN-γ, a cytokine of Th1, mRNA expression increased in PIA group, however it had no statistical difference comparing with control (*p* = 0.08). The other Th1 cytokine, TNF-α, was no changes between two groups (Fig 2A). Then we screened the mRNA expression level of Th2 cytokines. The expression of IL-4 and TGF-β increased in PIA group, and IL-10 expression level was significantly increased than control (Fig 2B). Interestingly, IL-17A, IL-21 and IL-22, Th17 cytokines, were significantly increased in PIA group. Specifically, IL-22 increased six folds than control and showed a remarkable increase in PIA group (Fig 2C). The IL-22 expression level enhanced gradually in the onset, acute and chronic phase of PIA model (data not shown). Clearly, the cytokines of Th17 were significant enhanced in the spleen of PIA rats.

### IL-22 expression of inguinal lymph nodes exhibited a remarkable increase in PIA

**Fig 3:**
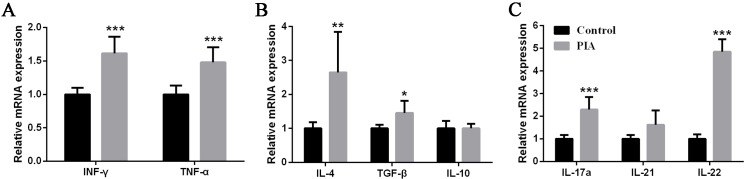
Expression changes of cytokines in the inguinal lymph nodes of PIA rats. **(A)** Expression of IFN-γ and TNF-α, Th1 cytokines, **(B)** expression of Th2 cytokines, IL-4, TGF-β and IL-10, and **(C)** expression of Th17 cytokines, IL-17A, IL-21 and IL-22, at 28 days in the inguinal lymph nodes from rats (n = 8 to 13 for each group) were measured by real-time qPCR. Relative mRNA expression was normalized by β-actin. Values are shown as means ± SEM. Levels of significance between the PIA group and control group were calculated by using Mann-Whitney U test (* *P*< 0.05, ** *P*< 0.01 and *** *P*< 0.001).

To investigate the change of Th1, Th2 and Th17 cytokines in other immune organs, and to confirm the increase of Th17 cytokines expression, we detected the expression of these cytokines in the inguinal lymph nodes at 28 days. IFN-γ and TNF-α, both cytokines of Th1, showed remarkable increase in PIA group, and had statistical difference between control group and PIA group (Fig 3A). IL-4 and TGF-β were significantly increased in PIA group, however the expression of IL-10 was not changed between two groups (Fig 3B). As the same with spleen, Th17 cytokines were increased in PIA group. IL-17A and IL-22 were significantly increased, however IL-21 has no statistical difference comparing with control (*p* = 0.056) (Fig 3C). Interestingly, IL-22 showed a remarkable increase as the same with the expression in spleen. Therefore, we concluded that IL-22 was significantly increased in the process of rat arthritis.

### The ratio of IL-22R1/IL-22BP exhibited a remarkable increase in PIA rat model

**Fig 4:**
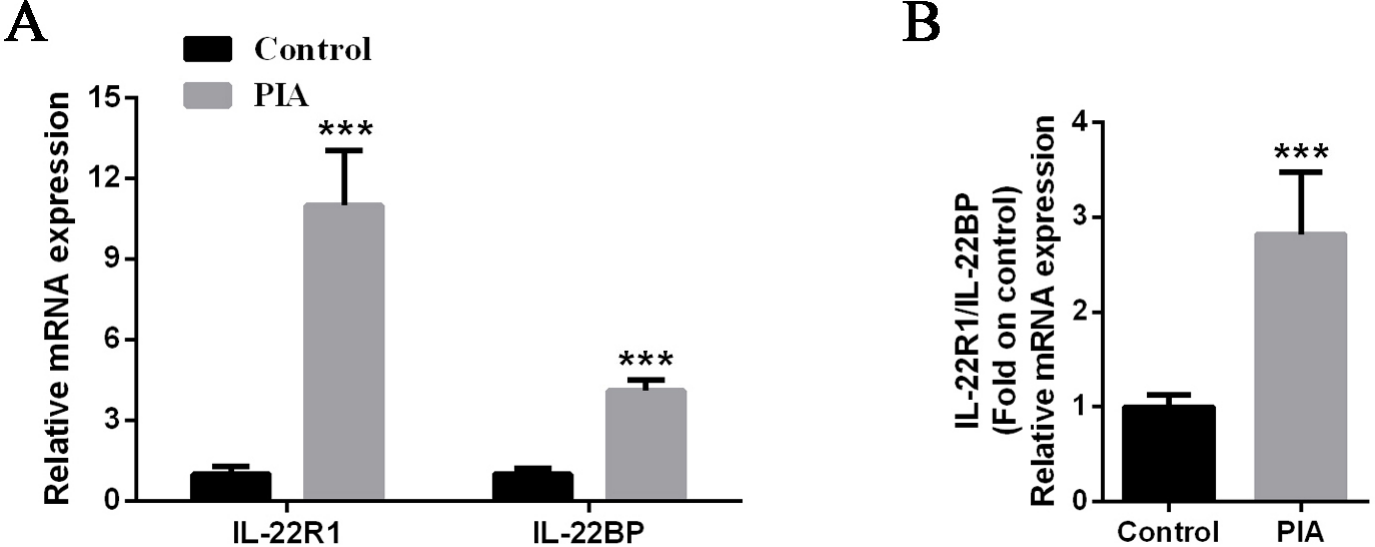
Expression level of IL-22 receptor and binding protein in the spleen of PIA rats. **(A)** Expression of IL-22R1 and IL-22BP at 28 days in the spleen from rats (n = 10 to 13 for each group) were measured by real-time qPCR. Relative mRNA expression was normalized by β-actin. **(B)** The ratio of IL-22R1/IL-22BP was evaluated from normalized expression. The IL-22R1/IL-22BP ratios were shown as means ± SEM. Levels of significance between the PIA group and control group were calculated by using Mann-Whitney U test (*** *P*< 0.001).

To confirm the expression level of IL-22 receptor and binding protein, two types of IL-22 receptors, in PIA rat model, we detected the mRNA expression of IL-22R1 and IL-22BP in spleen. The IL-22R1 and IL-22BP exhibited a remarkable increase in PIA group. Specifically, IL-22R1 increased ten folds than control group (Fig 4A). IL-22BP, a soluble protein, could competitively combine with IL-22 and IL-22R1, and inhibits IL-22 activity by blocking IL-22R1 [25]. The ratio of IL-22R1/IL-22BP was significantly increased and similar with IL-22 expression (Fig 4B). Thus, it is likely that signaling pathway of IL-22 was activated.

### IL-22 expression in synovial membrane and serum correlated with the severity of PIA

**Fig 5:**
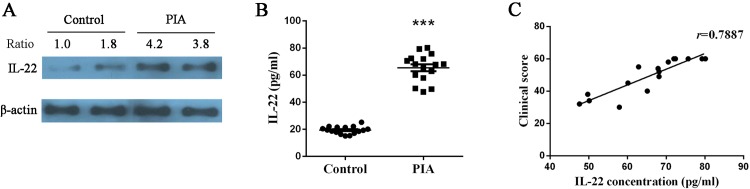
IL-22 expression in synovial membrane and serum correlated with the severity of PIA. **(A)** Expression of IL-22 protein at 28 days in the synovial membrane was measured by Western blotting. **(B)** Concentration of IL-22 protein at 28 days in the serum was detected by using ELISA. **(C)** Correlation between IL-22 concentration and clinical score was analyzed. One representative plot and quantitative data from three independent Western blotting experiments are shown. Ratio indicates the optical intensity of IL-22 protein bands against β-actin. IL-22 concentration shown are represented as means ± SEM. Levels of significance between the PIA group and control group were calculated by using Mann-Whitney U test (*** *P*< 0.001). Correlation was measured by Spearman’s σ analysis.

To address whether the enhancement of IL-22 is involved into disease development, we analyzed the correlation of IL-22 concentration in serum and clinical score, and the protein level of IL-22 in synovial membrane between two groups. The IL-22 expression of synovial membrane was detected by Western blotting, and results showed that PIA group had a significantly increased IL-22 expression (Fig 5A). The serum concentrations of IL-22 was analyzed in PIA and control rats, and 19.42 pg/ml in control group was lower than 65.47 pg/ml in PIA group (Fig 5B). The IL-22 concentration in PIA group correlated with clinical score (*r* = 0.7887) (Fig 5C). From these data we concluded that the expression of IL-22 in synovial membrane and serum was enhanced and correlated with the severity of PIA.

## Discussion

To sum up, PIA model in Dark Agouti rats led to chronic arthritis similar to RA in human. Our research implicated that cytokines of Th17 cells exhibited a remarkable increase than Th1 and Th2 cytokines in pathogenesis of PIA. Particularly, IL-22 was the most prominently increased cytokine which correlated with the severity of PIA. It is supposed that a significantly activated on the IL-22 signaling pathway playing a critical role in arthritis pathogenesis and IL-22 might aggravated the severity of PIA.

RA is a complex autoimmune disorder of which the pathogenic mechanism remains poorly understood. Although multiple genetic and environmental factors of RA are identified, it is unknown how the synovial inflammation occurs and perpetuates, and which types of cells are the most important inducers [26]. However, multiple cell types are involved in synovial inflammation. Endothelial activation can make leukocytes infiltrate synovial compartment by the expression of adhesion molecules and chemokines, which leads to synovitis occurrence [27]. T cells are abundant in synovial milieu, however T-cell-depleting therapeutics such as monoclonal antibodies directed against CD4, CD5, CD7 has shown efficacy but with limitation [28]. Hereby, it is possible that T cell subsets, Th cells for example, should play an important role.

Th cells are involved in host immunity by producing related cytokines, and Th1 cells are considered to be a drive of autoimmune disease. Although RA is conventionally considered to be a disorder which is mediated by Th1 cells, more attention has increasingly focused on Th17 cells which are potent inducers of tissue inflammation and have been associated with the pathogenesis of many experimental autoimmune disease and human inflammatory conditions [15, 29, 30]. In previous reports, Th17 cells and main effector cytokines were elevated in patients with RA, and the expression of IL-17 was also found to be considerable up-regulation in human synovial tissues of RA analyzing HG-U133A from Jena dataset [31–33]. Our results indicated that the expression of IL-17A, IL-21 and IL-22, Th17 cell cytokines, was increased in major immune organs of PIA rats. The increase of Th17 cells and their cytokines suggested their potential effect in arthritis development. Other previous studies on IL-17A has provided much evidence of Th17 cytokine as a pathogenic effector in RA and experimental arthritis. For instance, CIA was markedly suppressed in IL-17^-/-^ mice, and the production of antigen specific T cells and collagen specific immunoglobulin G2a were influenced [34]; IL-1 receptor antagonist-knockout (IL1rn^-/-^) Toll-like receptor 4-knockout (Tlr4^-/-^) mice was protected against severe arthritis and had markedly lower numbers of Th17 cells and a reduced capacity to produce IL-17 [35]; Th17 cells predominantly expressed CC chemokine receptor (CCR) 6, and migrated to the inflamed joints [36]. In addition, Th17 cells was critical for the onset phase of autoimmune arthritis, and secreted IL-17A acted on osteoblastic cells to differentiation by inducing RANKL [37].

Th17 cells are induced by CD4+ T cells in the presence of TGF-β and IL-21, and could produce IL-17, IL-17F, IL-21 and IL-22. These cytokines most likely cooperate to induce Th17-driven tissue inflammation. IL-21, IL-2 family cytokine, was first identified in 2000 and produced by activated T cells and NKT cells [38]. It has been described that IL-21 plays an important role in the expansion of previously activated B cells and in class switching of immunoglobulin isotypes [39]. The most amounts of IL-21 is produced by Th17 cells, and strongly induced by IL-6 [18, 40]. In addition, the effects of IL-21 depended on STAT3, but not on orphan nuclear receptor RORgammat which is a key specific transcription factor for Th17 cells [40]. In the arthritis research reports, blockade of IL-21R.Fc reduced the clinical and histologic signs of CIA [41]. As the results of our research, IL-21 showed an increase in PIA rats, but not remarkable changed than IL-17A and IL-22. We confirm that the main effects of IL-21 is inducing the differentiation of Th17 cells to produce other cytokines, and it might act in a positive feedback loop to amplify the Th17 responses and support tissue inflammation.

IL-22 is one of Th17 cytokine belonging to the IL-10 superfamily. The human *IL-22* gene is present on chromosome 12q15 in the vicinity of *IFN-γ* and *IL-26* genes [19]. Its production is promoted by IL-23, IL-1β, IL-7, Aryl Hydrocarbon Receptor (AhR) and Notch signaling. Specifically, one of the primary inducer of IL-22 production is IL-23, which is also secreted by granulocyte–macrophage colony-stimulating factor (GM-CSF) -induced antigen presenting cells and supports the differentiation and maintenance of Th17 cells in a feedback mechanism[42]. GM-CSF can induce granulocyte and macrophage populations from precursor cells, whose auto-antibody is a marker of aggressive Crohn’s disease[43, 44]. Because IL-22 receptor (IL-22R1) is not expressed in immune cells, but on epithelial and stromal cells including intestinal and respiratory epithelial cells, skin keratinocyte, hepatocytes, colonic subepithelial myofibroblasts, pancreatic acinar cells, and RA FLS(14,15). The main impact of IL-22 is on resident tissue cells, but not hematopoietic immune cells, and IL-22 not only have an effect on the regeneration of epithelial tissues following injury, but also on the host defense within barrier tissues [20]. It plays a dual role in different autoimmune disorders and animal models. The mouse model of psoriasis-like skin inflammation treated with IL-22-neutralizing antibodies could relieve inflammation, however treated with IL-22BP could aggravate disease in inflammatory bowel disease (IBD) model [45, 46]. Some researches show the relationship of IL-22 and arthritis animal models. In the IL-1Rα^-/-^ mice, anti-IL-22 treatment showed the reduction of clinical scores, inhibition of the amount of inflammatory cells and reduction of cartilage proteoglycan depletion[47]. Correspondingly, the IL-22^-/-^ mice showed less susceptible to CIA than wild-type mice with lower clinical scores, reduced arthritis severity and lower degree of pannus formation[48]. Similarly, in the CIA mice, anti-IL-22 treatment found lower joint inflammation scores than treated with isotype control, and lower cartilage destruction, bone involvement[49]. All these researches suggest that IL-22 played a proinflammatory role. While opposite evidence has also been found, which IL-22 played protective role in CIA mice model. Sarkar S. showed that IL-22 had a protective function with increased levels of IL-10 in CIA mice model when administered prior to the onset of the disease[22, 50]. These major differences are treating IL-22 in different time points of arthritis. One administrated anti-IL-22 at early stage (score range of 0–4), and the other treated IL-22 prior to the onset of arthritis. Similar phenomena have been showed in OX40L (or OX40 agonist) treated autoimmune disorders, which administration of OX40 agonist before the onset of the disease can increase Tregs and delay the onset of the disease, while treatment near onset day can exacerbate the disease[51]. In previous reports, IL-22^+^ CD4^+^ T cells are increased in the peripheral blood of patients with RA, and the level of IL-22 in serum is associated with radiographic progression of RA [31, 52]. And IL-22 promotes proliferation of RA FLS and expression of RANKL via p38 MAPK/NF-ΚB or JAK-2/STAT-3 signaling, leading to osteoclastogenesis [53]. IL-22 activated STAT3-dependent osteoblast-mediated bone remodeling[54]. IL-22 was involved in the proliferation, migration and osteogenic differentiation of mesenchymal stem cells[55]. All these studies show that IL-22 can also induce the osteoclastogenesis via promoting the expression of RANKL in RA FLS. Anti-IL-22 treatment significantly reduced the inflammation and bone erosion in IL-1Ra^-/-^ mice [47]. The parasitic worm-derived immunomodulator ES-62 protected against bone damage in CIA by suppressing IL-22[56]. Adiponectin injection resulted in an earlier onset of arthritis with bone erosion and high expression of IL-22 in CIA mice[57]. Although IL-22 is not directly related to the molecular mechanism of bone erosion, it could contribute to bone erosion. We found that IL-22 showed high expression in immune organs, synovial membrane and serum, and correlated with the severity of PIA. Collectively, these researches suggest that administration of IL-22 and other strategy, including type II collagen-driven anterior chamber-associated immune deviation (ACAID)-mediated immune tolerance via the generation of CD8^+^ Tregs, treated with GM-CSF or OX40L, could have therapeutic potential in context of RA[51, 58–60].

However, it is still unclear how IL-22 induce tissue inflammation and autoimmunity, and other biological functions of IL-22 in arthritis. It has been reported IL-22 promotes cells proliferation and enhances regeneration from tissue damage. And it seems that IL-22 plays a proinflammatory role in the pathogenesis of arthritis. These dual roles could lead to benefit and unprofitable results, and their mechanisms and relation are not known. Further researches should explorer underlying molecular mechanisms that IL-22 promotes proinflammation on synovial fibroblasts in RA.

## Conclusions

In summary, we identified differentially expressed cytokines in the spleen and inguinal lymph nodes of PIA rats, and found significant increase on mRNA levels of Th17 cytokines. Particularly, IL-22 was the most prominently increased cytokine. IL-22 expression was enhanced in synovial membrane and serum, and correlated with the severity of PIA rats. These findings are not only reveal a probably significant activated on the IL-22 signaling pathway in arthritis pathogenesis, but also provide important insights into other immunological disease. However, further researches are required to elucidate the molecular mechanism of IL-22 and other Th17 changed cytokines in the pathogenesis of PIA and RA.

## Supporting information

S1 Table NC3Rs ARRIVE guidelines checklist. (PDF)
